# Global DNA methylation variations after short-term heat shock treatment in cultured microspores of *Brassica napus* cv. Topas

**DOI:** 10.1038/srep38401

**Published:** 2016-12-05

**Authors:** Jun Li, Qian Huang, Mengxiang Sun, Tianyao Zhang, Hao Li, Biyun Chen, Kun Xu, Guizhen Gao, Feng Li, Guixin Yan, Jiangwei Qiao, Yongping Cai, Xiaoming Wu

**Affiliations:** 1 Oil Crops Research Institute of the Chinese Academy of Agricultural Sciences, Key Laboratory of Biology and Genetic Improvement of Oil Crops, Ministry of Agriculture, Wuhan 430062, P. R. China; 2 Department of Cell and Developmental Biology, College of Life Science, State Key Laboratory of Hybrid Rice, Wuhan University, Wuhan 430072, China; 3 Shanghai Greenglobe Biotechnology Co., Ltd, Shanghai 201499, P. R. China.

## Abstract

Heat stress can induce the cultured microspores into embryogenesis. In this study, whole genome bisulphite sequencing was employed to study global DNA methylation variations after short-term heat shock (STHS) treatments in cultured microspores of *Brassica napus* cv. Topas. Our results indicated that treatment on cultured Topas microspores at 32 °C for 6 h triggered DNA hypomethylation, particularly in the CG and CHG contexts. And the total number of T32 (Topas 32 °C for 6 h) vs. T0 (Topas 0 h) differentially methylated region-related genes (DRGs) was approximately two-fold higher than that of T18 (Topas 18 °C for 6 h) vs. T0 DRGs, which suggested that 32 °C might be a more intense external stimulus than 18 °C resulting in more changes in the DNA methylation status of cultured microspores. Additionally, 32 °C treatment for 6 h led to increased CHG differential methylations of transposons (DMTs), which were mainly constituted by overlaps between the hypomethylated differentially methylated regions (hypo-DMRs) and transposon elements (TEs). Further analysis demonstrated that the DRGs and their paralogs exhibited differential methylated/demethylated patterns. To summarize, the present study is the first methylome analysis of cultured microspores in response to STHS and may provide valuable information on the roles of DNA methylation in heat response.

Plants have evolved complicated genetic and epigenetic regulatory systems to respond quickly to unfavorable environmental conditions^1^. The alteration of growth patterns, through the adjustment of cell division and expansion, is a characteristic response of plants to environmental stress^2^. Plant reproduction, in particular pollen development, is the most stress-sensitive process in the life cycle of the organism^3^. Especially, developmental stages around the meiotic and mitotic divisions are the most vulnerable^4^,^5^. In angiosperms, microspores are generated by microsporocytes after meiosis and give rise to mature pollen after mitosis^6^. Increasing evidences showed that microspore as a specific cell type can deviate from the original gamete-producing pathway and enter into the embryogenesis after a short severe heat shock treatment^7^,^8^. And this heat treatment is often performed at 33–37 °C for a duration that varies from several hours to several days^8^. In *Brassica napus*, the most efficient temperature for microspore fate changing is obtained by increasing the culture temperature to 32 °C^9^,^10^. Using ultrastructural analysis, Telmer *et al*.^11^ demonstrated that the pollen differentiation pathway of the cultured microspores of *B. napus* cv. Topas is disrupted after short-term heat shock (STHS) treatment (within 6 h of 32.5 °C treatment). Apart from this, culture of isolated microspores of *B. napus* at 18 °C has been proposed as an ideal system to study the gametophytic development *in vitro*^10^. However, Prem *et al*.^12^ demonstrated that microspore embryogenesis (ME) induction in Topas could also be achieved at 18 °C with a longer duration, which should be extended to several weeks.

Along with its essential role in the maintenance of genome integrity, DNA methylation participates in regulation of genes which are significant for plant development and stress response^13^. Actually, external heat stimulation causes DNA methylation changes in plants^13^,^14^. A recent study has also demonstrated that DNA methylation was involved in the control of cell growth during heat stress in tobacco BY-2 cells^2^. Although heat pre-treatment is one of the significant stresses for ME induction^7^,^15^, few studies have examined whether DNA methylation changes in cultured microspores after STHS treatment. Here, we tried to employ genome-wide bisulphite sequencing (GWBS) to decipher global DNA methylation variations after 32 °C and 18 °C treatments for 6 h in cultured microspores of *B. napus* cv. Topas at single-base resolution. And our results revealed that 32 °C heat treatment for 6 h was sufficient to induce global DNA hypomethylation in cultured Topas microspores. And 32 °C might be a more intense external stimulus than 18 °C generating more changes in the DNA methylation status of cultured microspores. To summarize, the present study is the first methylome analysis of cultured microspores in response to STHS and may provide valuable information on the roles of DNA methylation in heat response.

## Results

### Microspore collection and culture

**Figure 1:**
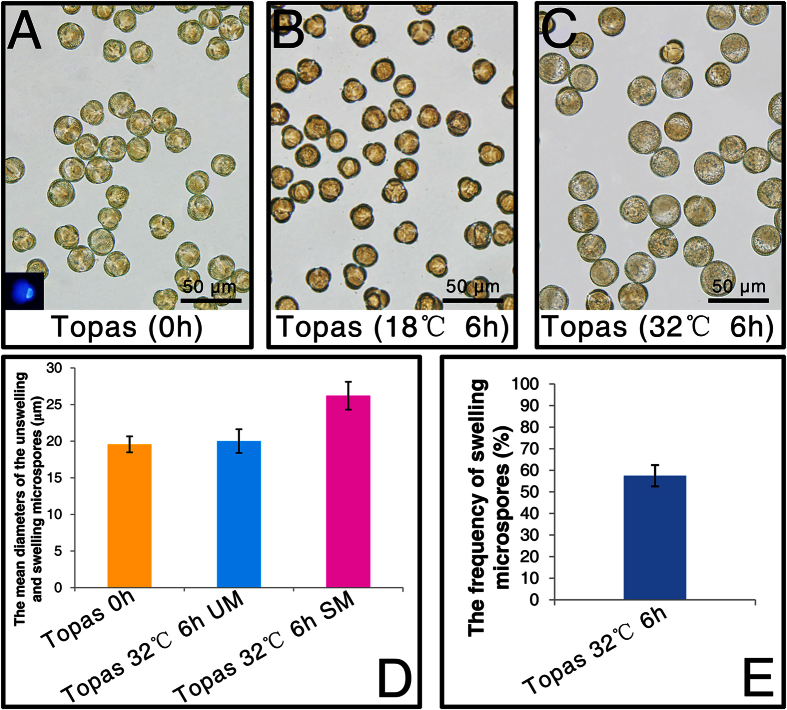
Microscope observation and statistical analysis of the heat treated Topas microspores *in vitro*. (**A**) 0 h microspores, (**B**) 18 °C 6 h treatment, (**C**) 32 °C 6 h treatment, (**D**) the mean diameters of swollen microspores (SM) and unswollen microspores (UM), (**E**) the frequency of SM. Small picture presenting in bottom left in (**A**) was the DAPI staining result.

A highly embryogenic cultivar Topas of *B. napus* was chosen for the analysis based on previous studies^8^,^16^. We attempted to collect only late uninucleate microspores as the initial materials for *in vitro* heat treatment and culture by bud selection and mesh screening (Fig. 1A). The mean diameter of these isolated microspores was 19.58 ± 1.09 μm (Fig. 1D). Then 32 °C and 18 °C treatments on the isolated microspores for 6 h were adopted in our experiments. Results showed that many enlarged microspores were formed after 6 h under 32 °C treatment rather than 18 °C treatment (Fig. 1B,C). The mean diameter and the frequency of the swollen microspores following the 32 °C treatment for 6 h were 26.22 ± 1.90 μm and 57.50 ± 7.50%, respectively (Fig. 1D,E). However, the mean diameter of the unswollen microspores under this same heat treatment condition was 20.02 ± 1.62 μm, which was almost identical to the size of the initial microspores (Fig. 1D). In order to decipher the global DNA methylation variations after treatments at 32 °C and 18 °C for 6 h in cultured microspores of *B. napus* cv. Topas, sample without treatment (Fig. 1A) was chosen as a control for DNA methylation comparisions.

### The DNA methylation landscapes of baseline and STHS-treated microspores

**Figure 2:**
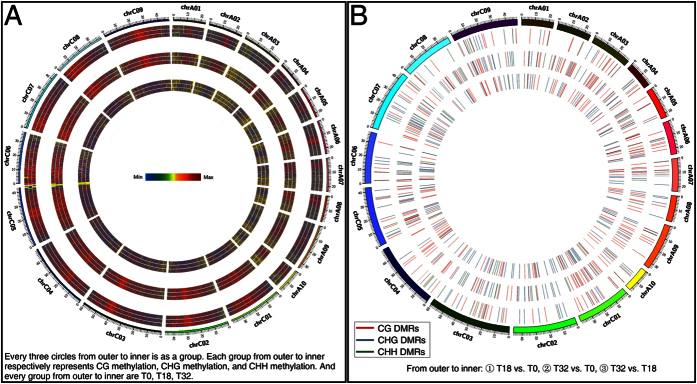
Heat map representation of methylated cytosines and Circos plot representation of DMRs.

Genomic DNA extracted from the three collected samples (T0, Topas 0 h; T18, Topas 18 °C for 6 h; T32, Topas 32 °C for 6 h) was treated with sodium bisulphite and then sequenced at ~26x coverage (Supplementary Table S1). The paired-end sequence files were subjected to multiple filtration steps and then aligned and deduplicated with Bismark (Supplementary Methods). The generated alignment reports indicated that the unique mapping efficiencies varied from 47.8% to 49.4% and the average proportions of methylation in three contexts (CG, CHG, and CHH) were 53.0%, 16.4%, and 3.5%, respectively (Supplementary Table S2). The level of symmetrical methylation was much higher compared with non-symmetrical methylation. Subsequent descriptive statistics by methylKit were shown in Supplementary Methods. Additionally, we depicted the identified methylated cytosines of the three samples on each chromosome (Fig. 2A). And the amount of identified methylated cytosines of the three samples distributed on the C genome was obviously higher than that distributed on the A genome (Fig. 2A).

### Identification of differentially methylated regions (DMRs)

**Figure 3:**
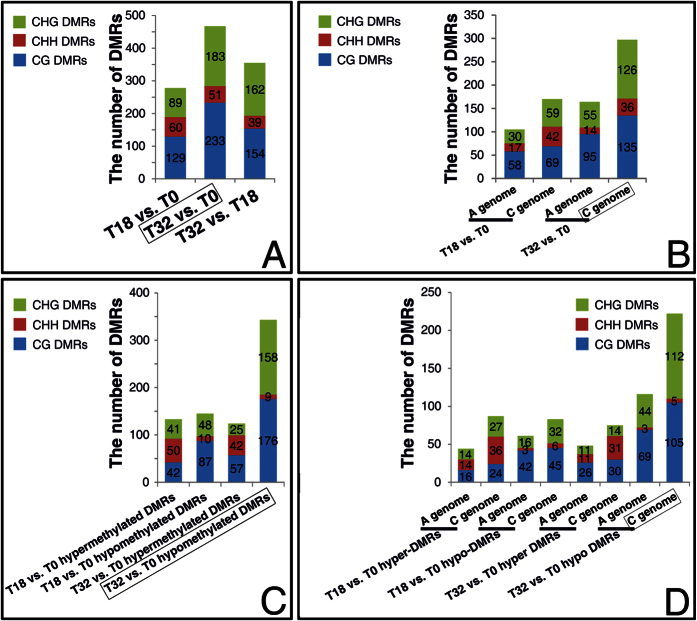
The number of DMRs.

**Figure 4:**
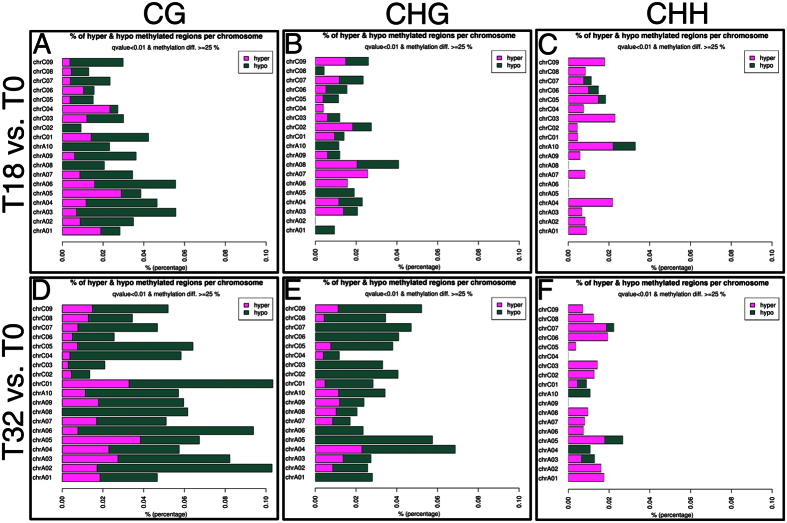
% of hyper and hypo methylated regions per chromosome.

To identify regions of the genome subjected to differential methylation, we used methylKit to calculate DMRs in a pairwise fashion (Fig. 2B and Supplementary Data S1). Then we randomly selected two DMRs for pyrosequencing validation, and the results confirmed the methylation status detected by GWBS (Supplementary Fig. S3). Deeper analysis indicated that more DMRs were identified in T32 vs. T0 than those in T18 vs. T0, which was mainly due to more CG and CHG DMRs were induced by 32 °C culture (Fig. 3A). Besides, more total DMRs were observed in the C genome than in the A genome in all pairwise samples (Fig. 3B and Supplementary Data S1), particularly in the T32 vs. T0 comparison (Fig. 3B). In T32 vs. T0 and T32 vs. T18 comparisons, there were more hypomethylated DMRs (hypo-DMRs) than hypermethylated DMRs (hyper-DMRs) (Fig. 3C and Supplementary Data S1). And these T32 vs. T0 and T32 vs. T18 hypo-DMRs were mainly occupied by CG and CHG DMRs (Fig. 3C and Supplementary Data S1). Moreover, 222 and 116 T32 vs. T0 hypo-DMRs were distributed on the C and A genomes, respectively (Fig. 3D). Analysis of the percentages of hyper- and hypo-methylated regions per chromosome indicated that the 32 °C treatment on the cultured Topas microspores for 6 h resulted in a significant proportion of hypomethylation in the symmetrical CG and CHG contexts (Fig. 4).

### Differential methylation in transposons

**Figure 5:**
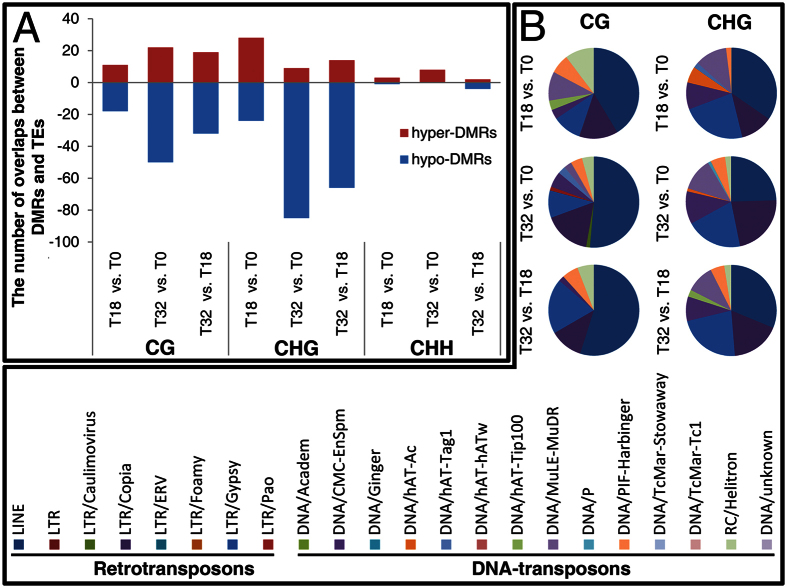
The analysis on the overlaps between DMRs and TEs.

Cytosine methylation is chiefly targeted towards transposon element (TE) silencing^17^. Stress-induced transposon activation has been confirmed by molecular data in many different hosts^14^,^18^,^19^. Therefore, it was necessary to investigate the overlapping information between the identified DMRs and TEs. Although Chalhoub *et al*.^20^ previously analysed the TEs identified in the released *B. napus* genome, their position information is not available in the public database. We firstly employed RepeatScout and RepeatMasker to *de novo* identify TEs in the whole *B. napus* genome. A total of 146,998 TEs (43,083 in the A genome and 103,915 in the C genome) were identified. Of them, 102,624 were retrotransposons and 44,374 were DNA transposons. Then, the differential methylations of transposons (DMTs) were searched based on the position information of TEs and DMRs (Supplementary Data S2). Further analysis showed that the amount of CHG DMTs was higher than the CG and CHH DMTs in T32 vs. T0 and T32 vs. T18 comparisons; the greater number of CHG DMTs was attributed to an increased overlap between the TEs and hypo-DMRs rather than hyper-DMRs (Fig. 5A, Supplementary Data S2, and Supplementary Table S4). Additionally, the differential methylations of retrotransposons (DMRTs) in all contexts in the three pairwise samples were more than the differential methylations of DNA transposons (DMDTs); LINE, LTR/Copia, and LTR/Gypsy were the most abundant retrotransposable elements (Fig. 5B and Supplementary Table S5). Subsequently, the distributions of DMTs on each chromosome were analysed. Results demonstrated that the numbers of DMTs located on A and C genomes were unequal (Supplementary Table S6).

### Identification of DMR-related genes (DRGs)

**Figure 6:**
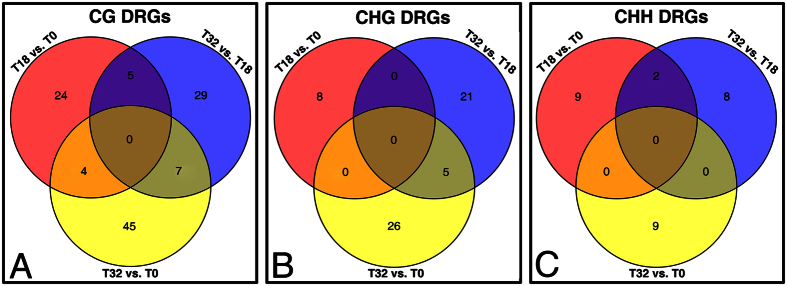
The overlaps of the identified DRGs among comparisons.

We employed the methylKit package and an in-house R script to identify DRGs (Supplementary Data S3). Then the distribution of DRGs on each chromosome of *B. napus* was investigated (excluding the DRGs located on unassembled scaffolds) (Supplementary Table S7). The total number of DRGs in T32 vs. T0 (96) was approximately two-fold higher than T18 vs. T0 (52), mainly because of the increase in CG and CHG DRGs in T32 vs. T0 (Fig. 6 and Supplementary Table S8). T32 vs. T0 DRGs could also be divided into 69 hypomethylated DRGs (hypo-DRGs) and 27 hypermethylated DRGs (hyper-DRGs), respectively. However, these two types of DRGs in T18 vs. T0 were almost equal (Table 1). Further analysis showed that only four common CG DRGs were identified between T32 vs. T0 and T18 vs. T0 (Fig. 6 and Table 2). They exhibited similar tendencies of methylation change in both comparisons (Table 2). Functional annotation analysis indicated that BnaA03g36810D was similar to AT3G22840, which encodes an early light-inducible protein (ELIP) (Table 2) and transiently accumulates in response to environmental stress^21^. Moreover, the mortality rates of plants lacking ELIPs are sometimes higher^22^. The *in vitro* culture itself was an environmental stress independent of the different temperatures. Did the culture rather than temperature effects induce the similar methylation changes of the common CG DRGs identified between T32 vs. T0 and T18 vs. T0?

To elucidate the differences of cultured microspores under different temperatures, subsequent analyses were mainly focused on the T32 vs. T18 DRGs (Table 2, Table 3 and Supplementary Data S3). Among the total 77 T32 vs. T18 DRGs, 47 were hypo-DRGs and 30 were hyper-DRGs (Table 1). In addition, five common CG and two common CHH DRGs were sought between T32 vs. T18 and T18 vs. T0, respectively (Fig. 6 and Table 2). Amazingly, the methylation/demethylation tendencies of these DRGs were totally opposite in two comparisons, and only BnaA03g24920D and BnaC05g07550D were hypo-DRGs in T32 vs. T18 (Table 2). BnaA03g24920D was similar to proton gradient regulation 5-like 1B (PGRL1B). And PGRL1 has been proved to be involved in cyclic electron flow (CEF), which only generated ATP and was driven by photosystems I (PSI)^23^. *Arabidopsis* PSI CEF is abolished following thermal-stress^24^. We next asked whether *PGRL1* maintained normal CEF functions during thermal-stress and generated sufficient energy for the survival of cultured microspores. BnaC05g07550D might encode LIM protein that regulating transcription or organizing the cytoskeleton by triggering the formation of actin bundles^25^. Actually, heat shock has been shown to cause changes in microtubule and cytoskeleton in cultured Topas microspores^26^.

All the seven common CG and five common CHG DRGs further identified between T32 vs. T18 and T32 vs. T0 had negative meth.diff values in both comparisons (Fig. 6 and Table 2). Among these DRGs, BnaA03g39290D was similar to *UBC29 (ubiquitin-conjugating enzyme 29*). UBCs participate in protein degradation via proteasome and may be involved in various biological processes^27^. As for the STHS treatment on cultured microspore can disrupt pollen differentiation^11^, we asked whether BnaA03g39290D functioned in sweeping the proteins that required for pollen differentiation pathway. Another common DRG BnaA04g24700D resembled *PDF1 (PROTODERMAL FACTOR 1*), which was involved in the fate determination of epidermal cell^28^. The CHG DRG BnaA06g03030D resembled a heat stress-responsive gene *FUT12 (fucosyltransferase 12*)^29^. In addition, BnaC01g24140D was similar to *AtDOK1 (A. thaliana dolichol kinase 1*), the expression of which could complement the temperature-sensitive growth and glycosylation defects of the *Saccharomyces cerevisiaesec59* mutant^30^. Moreover, AtDOK1 is involved in the synthesis of dolichol phosphate (Dol-P), which serves as a carrier of complex polysaccharides during protein glycosylation^30^. Glycoproteins are confirmed to be involved in adaptation to biotic and abiotic stresses^31^.

Except for the common DRGs, there were 58 specific DRGs (33 hypo-DRGs and 25 hyper-DRGs) existing in T32 vs. T18, including 29 CG, 21 CHG, and eight CHH DRGs (Table 3). These specific DRGs contained several genes that might be involved in various biological processes (Table 3). For example, BnaA08g05750D (thioredoxin superfamily protein) and BnaC02g29760D (glutathione S-transferase family protein) might function in maintaining cell redox homeostasis (Table 3). High temperature provokes the accumulation of reactive oxygen species (ROS) in plants^32^. Heat stress-incuded ME also generates an oxidative burst and ROS^33^. Therefore, sustaining cell redox homeostasis was crucial for the survival of cultured microspore during heat stress. Additionally, two putative glycosyltransferases (BnaC06g12760D and BnaC06g04480D) and a putative fasciclin-like arabinogalactanprotein 2 (FLA2) (BnaA06g19130D) were found as specific hypo-DRGs in T32 vs. T18 (Table 3). Previous study has indicated that the protein encoded by AT5G39990 (similar to BnaC06g12760D) was involved in the biosynthesis of type II arabinogalactan (AG) and cell elongation during seedling growth^34^. Most AGPs are *O*-glycosylated at hydroxyproline residues by type II AG group^35^. And their pivotal roles in cell wall signal transduction, plant development and stress tolerance have also been discussed^36^. Intriguingly, *FLA2* was found to be significant in several stress-related AFGC microarray experiments^37^. It could be part of the auxin transporters ABCB19/PINFORMED1 (PIN1) nanodomain^38^. In addition, BnaC03g42260D resembled *AtSGP2 (A. thaliana* G-protein) (Table 3). *Arabidopsis* monomeric G-proteins are implied to be markers of early and late events in cell differentiation^39^. Intriguingly, a specific CHG hypo-DRG BnaC01g19320D coincidently matched with napin gene *SESA4 (seed storage albumin 4*) (Table 3), which was identified as an early molecular marker for ME in *B. napus*^16^,^40^. Another specific CHG hypo-DRG BnaC01g21110D identified from T32 vs. T18 might be a putative *SCL (scarecrow-like*) gene (Table 3). Joosen *et al*.^9^ previously identified a *B. napus* gene, which was homologous to *Arabidopsis SCL11*, as a robust marker for ME. Whether these STHS-induced DRGs are really related to ME is still an open question. Besides, other gene groups related to energy metabolism, protein degradation, transcription and translation, and signal transduction (Table 3) were also included in specific T32 vs. T18 DRGs.

### Chromosome localization of DRGs and their paralogs

**Figure 7:**
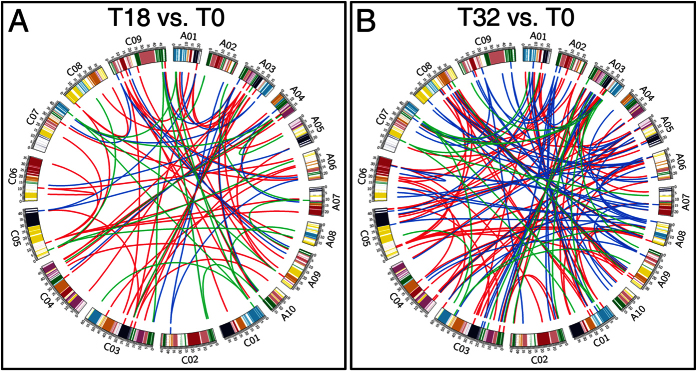
Circos plot of the DRGs and their corresponding paralog genes. The colors displayed in chromosomes represented A-X CCBs. The paralog gene pairs were connected by lines. And the red, blue, and green lines stranded for CG, CHG, and CHH DRGs, respectively. Similarly, red, blue, and green tickets along with the inner chromosomes indicated the locations of CG, CHG and CHH DRGs on chromosomes, respectively.

Schranz *et al*.^41^ have built the A to X conserved collinear blocks (CCBs) of the reduced karyotype (n = 5) of *A. thaliana*, and these blocks were defined by their position in a proposed ancestral karyotype (n = 8). The genomes of *Brassica rapa* (AA) and its sister species *Brassica oleracea* (CC) were almost complete triplications of the genome of *A. thaliana*^42^,^43^. *B. napus* (AACC) was formed by recent allopolyploidy between the ancestors of *B. oleracea* and *B. rapa*^20^,^44^. Therefore, the CCBs of *B. napus* were constructed based on the position information of the *A. thaliana* A to X segments^41^. Then we investigated the number of DRGs in each of the A to X CCBs of *B. napus*. Results indicated that the U and F blocks possessed the most DRGs in the T32 vs. T0 and T18 vs. T0 comparisons, whereas no DRGs were distributed on the G, K, and S blocks (Supplementary Table 9). Due to the polyploid properties, it was also necessary to evaluate whether differential methylated An-Cn paralog gene pairs existed in *B. napus*. The An-Cn paralog gene pairs of *B. napus* were searched according to the methods described by Liu *et al*.^43^. Although almost all of the T32 vs. T0 and T18 vs. T0 DRGs possessed one or more paralog genes, none of these paralog genes were included in the identified DRGs (Fig. 7 and Supplementary Data 4). This result suggested that distinct DNA methylation regulatory pathways might exist even for paralogs. Chang and Liao^45^ demonstrated that DNA methylation could “rebalance” the overall expression dosage of paralogs.

### Real-time PCR to analyse DRG expression

**Figure 8:**
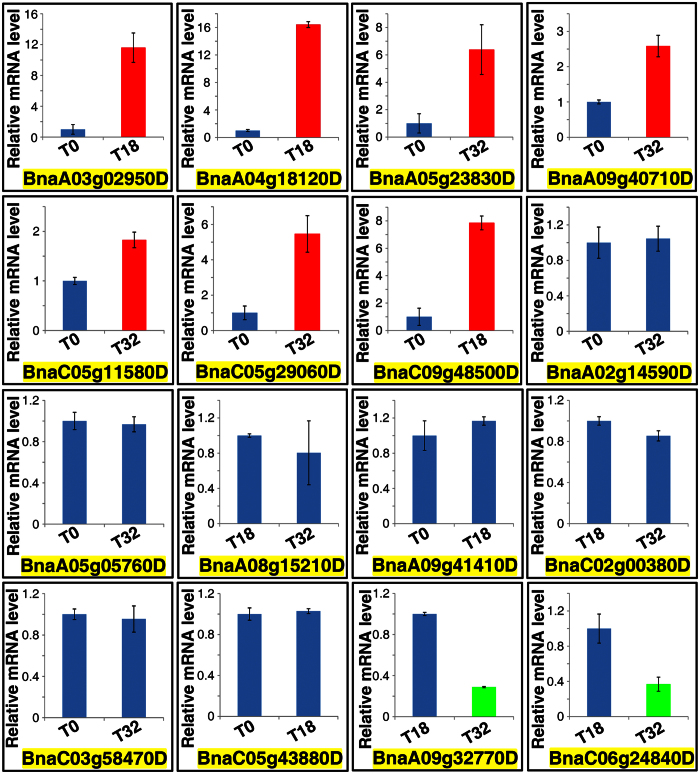
Quantitative RT-PCR analysis of the 16 randomly selected DRGs.

To examine the relationships between DNA methylation and gene expression levels, 16 DRGs (13 hypo-DRGs and three hyper-DRGs) were randomly selected for real-time PCR verification based on the UniGene information (Supplementary Data S5). Results indicated that seven genes were up regulated, two genes were down regulated, and the expression levels of seven genes remained unchanged (Fig. 8). DNA demethylation and methylation are considered to be associated with elevating and suppressing gene expressions, respectively. However, the expressions of four hypo-DRGs and two hyper-DRGs did not follow this principle (Fig. 8 and Table 4). More dynamic and complex relationships between DNA methylation and expression have been illustrated in other studies^46^,^47^. Among these 16 selected genes, BnaA09g40710D was a T32 vs. T0 CG hypo-DRG and its expression was up-regulated by nearly two-fold in T32 compared with that of T0 (Fig. 8). Furthermore BnaA09g40710D was identical to *Arabidopsis ELF3 (early flowering 3*) (Table 4), which controlled elongation growth in response to temperature^48^. Similarly, another hypo-DRG BnaC05g29060D was up-regulated in the 32 °C treatment (Fig. 8). This gene resembled *Arabidopsis SCAMP5 (secretory carrier membrane protein 5*) (Table 4). Whether BnaC05g29060D functions in vesicles transportation and protein trafficking during heat treatment remains unknown.

## Discussion

Exposure of Arabidopsis plants to heat stress results in an increased global methylation^49^. Nevertheless, in cotton (*Gossypium hirsutum*) anthers, high temperature leads to the genome-wide hypomethylation at the tetrad stage and the tapetal degradation stage^50^. Our results showed that the T32 vs. T0 and T32 vs. T18 DMRs were mainly comprised of hypo-DMRs rather than hyper-DMRs (Fig. 3C and Supplementary Data S1). The percentages of the hyper- and hypo-methylated regions per chromosome clearly indicated that the 32 °C treatment on cultured microspores generated a significant proportion of hypomethylation in the symmetrical CG and CHG contexts (Fig. 4). This result demonstrated that STHS initiated the demethylation process, giving rise to a decrease in global DNA methylation in cultured microspores. Actually, the percentage of methylated cytosine increased from 3 to about 11% from microspore to mature stage in pollen of *B. napus*^51^. Therefore, it seems that there is no consistent trend in the changes of DNA methylation under heat in different species or in different cell types. Whether plant male germ cell tended to be global hypomethylated rather than hypermethylated after heat stress? If so, why the DNA methylation tendencies were different? Additionally, we also found that the total number of DRGs in T32 vs. T0 was approximately two-fold higher than T18 vs. T0 suggesting 32 °C may be a more intense external stimulus than 18 °C and ultimately resulted in more changes in the DNA methylation status of cultured microspores (Table 1 and Fig. 6).

In plants, DNA methylation occurs frequently in all three sequence contexts: the symmetric CG and CHG contexts and the asymmetric CHH context. Each type of DNA methylation is vital for development and responses to environmental stresses^52^. Here, our data revealed that the 32 °C treatment brought about increased CHG DMTs in the T32 vs. T0 and T32 vs. T18 comparisons due to abundant overlaps between the TEs and hypo-DMRs (Fig. 5A). Moreover, DMRTs in all contexts in the three pairwise samples were higher than DMDTs. LINE, LTR/Copia, and LTR/Gypsy were the most abundant retrotransposable elements (Fig. 5B and Supplementary Table S5). Yang *et al*.^47^ previously observed that retrotransposons were more tightly controlled by methylation than DNA transposons during the floral development of *Arabidopsis*. ONSEN, an LTR/copia type retrotransposon was found activated by heat stress in *Arabidopsis*^18^,^19^. Deeper studies are needed to answer whether and how these DMRTs function during STHS treatment in cultured microspores.

Intriguingly, some molecular markers, such as napin, G-protein, *SCL* and AGP, for early ME in *B. napus* identified via transcriptome and proteome analysis were also existed among the 58 specific T32 vs. T18 DRGs (Table 3). Additional experiments are required to decipher how DNA methylation functions as an epigenetic response to heat stress in cultured microspores, whether DNA methylation status is associated with the expression of the overlapped markers for early ME in *B. napus* and whether the micropsores from different cultivars that with different embryogenic potentials possess different epigenetic response to heat stress.

## Methods

### Sample preparation, GWBS, and genome-wide cytosine methylation analysis

Plants of the *B. napus* inbred line ‘Topas’ were sown in an experimental field at Qinghai University (Xining, Qinghai Province, China) on April 10, 2014 (Supplementary Methods). Bud selection, microspore isolation and cultivation were performed (Supplementary Methods). Genomic DNA and total RNA were extracted for further analysis (Supplementary Methods). Qualified genomic DNA was sent to BGI (BGI Tech Solutions Co., Ltd, Shenzhen, China) for GWBS (Supplementary Methods). Genome-wide cytosine methylation analysis was carried out based on the methods described in Supplementary Methods. Two randomly selected regions that contained identified ^m^C sites were used to perform pyrosequencing validation (Supplementary Methods). Additionally, we performed CG island prediction and digital expression analyses (Supplementary Methods).

### Analysis of the overlaps between TEs and DMRs

First, RepeatScout (http://bix.ucsd.edu/repeatscout/) was used to construct a repeat library of the *B. napus* genome using an *ab initio* approach. Then this library was used to screen DNA sequences for interspersed repeats and low complexity DNA sequences using RepeatMasker (http://www.repeatmasker.org/). Retrotransposon and DNA transposons position information was retrieved from the out file generated by RepeatMasker. An in-house Perl script was used to calculate the overlaps between TEs and DMRs.

### Quantitative RT-PCR analysis

Gene-specific primers (Supplementary Table S10) for the 16 randomly selected DRGs were designed with GeneTool. The housekeeping gene *β-actin* (AF111812) was used to normalize the expression of each gene in the different RNA samples. cDNA synthesis and quantitative RT-PCR analysis was performed according to the methods described by Li *et al*.^53^.

## Additional Information

**How to cite this article**: Li, J. *et al*. Global DNA methylation variations after short-term heat shock treatment in cultured microspores of *Brassica napus* cv. Topas. *Sci. Rep.* **6**, 38401; doi: 10.1038/srep38401 (2016).

**Publisher's note:** Springer Nature remains neutral with regard to jurisdictional claims in published maps and institutional affiliations.

## Supplementary Material

Supplementary Information

Supplementary Dataset 1

Supplementary Dataset 2

Supplementary Dataset 3

Supplementary Dataset 4

Supplementary Dataset 5

## Figures and Tables

**Table 1:** The number of Hypo/Hyper-DRGs identified in three pairwise comparisons.

	T18 vs. T0	T32 vs. T0	T32 vs. T18
Hypo-DRGs	28	69	47
Hyper-DRGs	24	27	30
Total DRGs	52	96	77

**Table 2:** The common DRGs identified between comparisons.

Comparisons	TD^a^	DRG Locus	From	MDD^b^	DDT^c^	S^d^	SAL^e^	Description (TIGR)
T32 vs. T18 & T32 vs. T0	CG hypo-DRGs	BnaA02g21500D	T32 vs. T18	−31.31	0	+	AT4G22758	unknown protein
			T32 vs. T0	−30.94				
		BnaA03g39290D	T32 vs. T18	−37.09	−138	+	AT2G16740	ubiquitin-conjugating enzyme 29 (UBC29)
			T32 vs. T0	−36.11				
		BnaA03g54520D	T32 vs. T18	−30.61	0	−	AT4G04090	BTB/POZ domain-containing protein
			T32 vs. T0	−27.20				
		BnaA04g24700D	T32 vs. T18	−39.49	−1873	−	AT2G42840	protodermal factor 1 (PDF1)
			T32 vs. T0	−35.25				
		BnaC03g23920D	T32 vs. T18	−36.65	−110	−	ATCG00190	RNA polymerase subunit beta, RPOB
			T32 vs. T0	−28.17				
		BnaC08g29000D	T32 vs. T18	−47.18	−1363	−	AT3G58120	BZIP transcription factor 61 (BZIP61)
			T32 vs. T0	−38.19				
		BnaC09g13630D	T32 vs. T18	−30.31	0	−	NA^f^	NA
			T32 vs. T0	−25.65				
	CHG hypo-DRGs	BnaA02g16440D	T32 vs. T18	−26.83	−1324	+	NA	NA
			T32 vs. T0	−27.68				
		BnaA06g03030D	T32 vs. T18	−27.65	−975	−	AT1G49710	fucosyltransferase 12 (FUT12)
			T32 vs. T0	−33.45				
		BnaC01g24140D	T32 vs. T18	−34.33	−1673	−	AT3G45040	dolichol kinase 1 (DOK1)
			T32 vs. T0	−28.92				
		BnaC03g31070D	T32 vs. T18	−38.24	−1693	−	AT4G02110	transcription coactivators
			T32 vs. T0	−54.76				
		BnaC06g31320D	T32 vs. T18	−27.14	−1585	+	AT1G70230	altered xyloglucan 4 (AXY4)
			T32 vs. T0	−29.29				
T32 vs. T0 & T18 vs. T0	CG hypo-DRGs	BnaA02g21210D	T18 vs. T0	−49.26	0	−	NA	NA
			T32 vs. T0	−48.31	0			
				−43.61	−66			
		BnaA03g36810D	T18 vs. T0	−29.68	9	−	AT3G22840	early light-inducible protein 1 (ELIP1)
			T32 vs. T0	−33.98	1009			
		BnaC03g43830D	T18 vs. T0	−31.55	383	−	NA	NA
			T32 vs. T0	−29.22				
	CG hyper-DRGs	BnaC03g57230D	T18 vs. T0	35.03	1399	−	AT3G43610	Spc97/Spc98 family of spindle pole body (SBP) component
			T32 vs. T0	37.62				
T32 vs. T18 & T18 vs. T0	CG DRGs	BnaA03g17310D	T32 vs. T18	32.38	51	−	AT2G37600	ribosomal protein L36e family protein
			T18 vs. T0	−29.80				
		BnaA03g24920D	T32 vs. T18	−30.84	−382	−	AT4G11960	proton gradient regulation 5-like 1B (PGRL1B)
			T18 vs. T0	37.52				
		BnaA03g38010D	T32 vs. T18	30.91	0	+	AT2G05160	CCCH-type zinc finger family protein with RNA-binding domain
			T18 vs. T0	−32.58				
		BnaA04g16720D	T32 vs. T18	40.20	−1291	−	AT2G28830	plant U-box 12 (PUB12)
			T18 vs. T0	−26.29				
		BnaC05g34070D	T32 vs. T18	25.39	0	−	NA	NA
			T18 vs. T0	−27.71				
	CHH DRGs	BnaC05g07550D	T32 vs. T18	−32.10	640	−	AT1G10200	ATWLIM1
			T18 vs. T0	35.71				
		BnaC07g43260D	T32 vs. T18	64.58	−152	+	AT4G31340	myosin heavy chain-related
			T18 vs. T0	−32.49				

^a^TD, type of DRG.

^b^MDD, meth.diff of the DMR.

^c^DDT, distance from DMR to transcriptional start site.

^d^S, strand.

^e^SAL, similar to Arabidopsis locus.

^f^NA, no available.

**Table 3:** The specific DRGs identified in T32 vs. T18.

TD^a^	MDD^b^	DRG Locus	DDT^c^	S^d^	SAL^e^	Description (TIGR)
CG hypo-DRGs	−40.67	BnaA03g39270D	0	+	NA^f^	NA
	−27.90	BnaA09g46290D	−87	−	AT1G13410	Tetratricopeptide repeat (TPR)-like superfamily protein
	−31.82	BnaC01g25270D	0	−	NA	NA
	−27.24	BnaC01g33320D	1906	−	NA	NA
	−25.69	BnaC01g35440D	0	+	NA	NA
	−25.95	BnaC03g44230D	−1048	+	AT2G04520	Nucleic acid-binding, OB-fold-like protein
	−25.89	BnaC06g00730D	−1195	+	NA	NA
	−25.61	BnaC07g12960D	1789	−	NA	NA
	−28.44	BnaC08g34040D	0	−	NA	NA
	−29.39	BnaA01g13320D	478	−	AT4G23640	tiny root hair 1 (TRH1)
	−26.97	BnaA07g06910D	1253	−	AT1G31320	lob domain-containing protein 4 (LBD4)
	−26.57	BnaA08g02650D	411	+	AT1G49480	related to vernalization1 1 (RTV1)
	−29.10	BnaA08g02660D	724	−	AT1G49475	AP2/B3-like transcriptional factor family protein
	−34.68	BnaA08g05750D	1395	+	AT4G15660	thioredoxin superfamily protein
	−36.21	BnaC04g33690D	−1177	+	AT2G21170	triosephosphate isomerase (TIM)
	−36.54	BnaC07g08220D	310	−	AT4G14360	S-adenosyl-L-methionine-dependent methyltransferases superfamily protein
CG hyper-DRGs	34.20	BnaA03g49000D	183	+	NA	NA
	35.27	BnaA03g39020D	377	+	AT2G16365	F-box family protein
	46.27	BnaA05g10770D	626	+	AT2G32280	vasculature complexity and connectivity (VCC)
	26.27	BnaA05g16350D	−1480	+	AT1G32180	cellulose synthase-like d6 (CSLD6)
	28.30	BnaA07g12210D	1222	−	AT5G66985	unknown protein
	40.63	BnaA09g20620D	−342	−	AT4G04020	plastoglobulin 35 (PGL35)
	29.73	BnaC01g08880D	0	−	AT4G29660	embryo defective 2752 (EMB2752)
	26.54	BnaC03g25860D	0	+	AT2G46530	auxin response factor 11 (ARF11)
	38.37	BnaC04g03320D	−1631	−	AT2G44090	ankyrin repeat family protein
	50.37	BnaC04g39580D	−1581	+	AT2G28190	copper/zinc superoxide dismutase 2 (CSD2)
	33.33	BnaC05g08160D	420	−	AT1G10650	SBP (S-ribonuclease binding protein) family protein
	26.85	BnaC06g24840D	−845	−	AT1G70180	Sterile alpha motif (SAM) domain-containing protein
	32.29	BnaC07g08840D	1196	+	AT1G30550	S-adenosyl-L-methionine-dependent methyltransferases superfamily protein
CHG hypo-DRGs	−32.48	BnaA06g19130D	−1177	−	AT4G12730	FASCICLIN-like arabinogalactan 2 (FLA2)
	−33.36	BnaC01g20920D	−467	−	NA	NA
	−43.07	BnaC03g04740D	879	+	NA	NA
	−28.40	BnaC08g28690D	0	−	NA	NA
	−26.83	BnaC01g19320D	−1456	+	AT4G27170	seed storage albumin 4 (SESA4)
	−25.67	BnaC01g21110D	−1848	+	AT4G17230	scarecrow-like 13 (SCL13)
	−42.05	BnaC02g29760D	1635	+	AT5G42150	glutathione S-transferase (GST) family protein
	−26.87	BnaC03g01150D	1557	+	AT5G03330	cysteine proteinases superfamily protein
	−27.27	BnaC03g23710D	−1234	−	AT2G42380	BZIP transcription factor 34 (BZIP34)
	−30.15	BnaC03g42260D	−1429	+	AT3G21700	*Arabidopsis thaliana* G protein (AtSGP2)
	−34.65	BnaC03g65030D	−428	−	AT4G22570	adenine phosphoribosyl transferase 3 (APT3)
	−35.83	BnaC06g12760D	−505	+	AT5G39990	beta-glucuronosyltransferase 14a (GLCAT14A)
	−25	BnaC07g42330D	0	−	AT4G30080	auxin response factor 16 (ARF16)
	−28.69	BnaC09g29070D	−1151	+	AT5G53030	unknown protein
CHG hyper-DRGs	25.59	BnaA09g10660D	14	−	NA	NA
	43.44	BnaA01g13520D	−1153	+	AT4G23900	nucleoside diphosphate kinase family protein
	40.28	BnaA04g17380D	−1063	−	AT2G30210	laccase 3 (LAC3)
	26.05	BnaA08g15210D	−115	−	AT4G36350	purple acid phosphatase 25 (PAP25)
	27.29	BnaC02g00380D	−284	+	AT5G65360	histone 3.1 (H3.1)
	29.43	BnaC03g19880D	−1382	−	AT2G36020	hva22-like protein j (HVA22J)
	32.60	BnaC05g39140D	−1220	−	AT3G14450	ctc-interacting domain 9 (CID9)
CHH hypo-DRGs	−25.65	BnaA09g32770D	−873	+	AT3G52300	“ATP synthase d chain, mitochondrial” (ATPQ)
	−25.15	BnaC01g04970D	−1207	+	AT4G33210	slow motion (SLOMO)
	−28.54	BnaC06g04480D	−490	+	AT1G51210	UDP-Glycosyltransferase superfamily protein
CHH hyper-DRGs	25.05	BnaC02g26820D	−329	−	NA	NA
	31.20	BnaC01g10210D	903	+	AT4G17615	calcineurin b-like protein 1 (CBL1)
	37.82	BnaC05g05560D	719	+	AT1G07900	lob domain-containing protein 1 (LBD1)
	26.88	BnaC05g39760D	−1671	+	AT3G13560	*O*-Glycosyl hydrolases family 17 protein
	27.14	BnaC09g32910D	0	−	AT5G57320	villin 5 (VLN5)

^a^TD, type of DRG.

^b^MDD, meth.diff of the DMR.

^c^DDT, distance from DMR to transcriptional start site.

^d^S, strand.

^e^SAL, similar to *Arabidopsis* locus.

^f^NA, no available.

**Table 4:** The relationships between DNA methylation variations and expression levels of the 16 selected DRGs.

DRG Locus	SAL^a^	Description (TIGR)	Comparison	SCD^b^	DDT^c^	MDD^d^	Expression	Consistency
BnaC09g48500D	AT5G07290	mei2-like 4 (ML4)	T18 vs. T0	CG	−150	−49.48	up	consistent
BnaA03g02950D	AT5G10480	pasticcino 2 (PAS2)	T18 vs. T0	CHG	−454	−25.43	up	consistent
BnaA02g14590D	NA^e^	NA	T32 vs. T0	CG	0	29.33	down	consistent
BnaA09g40710D	AT2G25930	early flowering 3 (ELF3)	T32 vs. T0	CG	−519	−46.84	up	consistent
BnaC05g11580D	AT1G15280	NA	T32 vs. T0	CG	−935	−26.71	up	consistent
BnaA05g23830D	NA	NA	T32 vs. T0	CHG	−482	−36.91	up	consistent
BnaC05g29060D	AT1G32050	secretory carrier membrane protein 5 (SCAMP5)	T32 vs. T0	CHG	−251	−39.48	up	consistent
BnaC03g58470D	AT1G28260	telomerase activating protein Est1	T32 vs. T0	CHH	0	71.43	down	consistent
BnaA08g15210D	AT4G36350	purple acid phosphatase 25 (PAP25)	T32 vs. T18	CHG	−115	26.05	down	consistent
BnaC02g00380D	AT5G65360	histone 3.1 (H3.1)	T32 vs. T18	CHG	−284	27.29	down	consistent
BnaA09g41410D	AT2G24420	DNA repair ATPase-related	T18 vs. T0	CG	−807	−33.65	down	contradictory
BnaC05g43880D	AT3G08680	leucine-rich repeat protein kinase family protein	T18 vs. T0	CHG	−396	−32.82	down	contradictory
BnaA04g18120D	AT2G31305	inhibitor-3 (INH3)	T18 vs. T0	CHH	−3	28.79	up	contradictory
BnaA05g05760D	AT2G39800	delta1-pyrroline-5-carboxylate synthase 1 (P5CS1)	T32 vs. T0	CHG	−335	−27.57	down	contradictory
BnaC06g24840D	AT1G70180	sterile alpha motif (SAM) domain-containing protein	T32 vs. T18	CG	−845	26.85	unchanged	contradictory
BnaA09g32770D	AT3G52300	“ATP synthase D chain, mitochondrial” (ATPQ)	T32 vs. T18	CHH	−873	−25.65	unchanged	contradictory

^a^SAL, similar to *Arabidopsis* locus.

^b^SCD, sequence context of DMR.

^c^DDT, distance from DMR to transcriptional start site.

^d^MDD, meth.diff of the DMR.

^e^NA, no available.
