# Supplementary material for: Global DNA methylation variations after short-term heat shock treatment in cultured microspores of Brassica napus cv. Topas
Source: Sci Rep. 2016 Dec 5;6:38401. doi: 10.1038/srep38401 (PMC5137020; doi:10.1038/srep38401)
Supplement: Supplementary Information [file srep38401-s1.pdf]

Global DNA methylation variations after short-term heat shock treatment in cultured microspores of *Brassica napus* cv. Topas

Jun Li[1], Qian Huang[1], Mengxiang Sun[2], Tianyao Zhang[1], Hao Li[1], Biyun Chen[1], Kun Xu[1], Guizhen Gao[1], Feng Li[1], Guixin Yan[1], Jiangwei Qiao[1], Yongping Cai[3] & Xiaoming Wu[1*]

[1]Oil Crops Research Institute of the Chinese Academy of Agricultural Sciences, Key Laboratory of Biology and Genetic Improvement of Oil Crops, Ministry of Agriculture, Wuhan 430062, P. R. China

[2]Department of Cell and Developmental Biology, College of Life Science, State Key Laboratory of Hybrid Rice, Wuhan University, Wuhan 430072, China

[3]Shanghai Green Globe Biotechnology Co., Ltd

Author for correspondence: *Xiaoming Wu*[*]

Tel: +86-027-86812906

Email: wuxm@oilcrops.cn

Supplementary Methods

Plant Materials and growth conditions

Prior to seeding, the soil of the experimental field at Qinghai University (Xining, Qinghai Province, China) was plowed twice and leveled, and chemical fertilizers were applied at the rate of 750 kg/ha using an N-P_2O_5-K_2O formula at 15-15-15. The *B. napus* inbred lines 'Topas' was sowed on April 10, 2014 with 1/3 m row spacing. At 40 days post-emergence, the distance between individual plants in each row was manually thinned to 0.15 m. Individual plant in each plot that did not show any obvious phenotypic differences were selected and kept at several developmental stages, to avoid the erroneous inclusion of plants not belonging to the plot.

Confirmation of the correlation between the bud lengths and the developmental stages of microspores

As for the developmental stage of microspores is one of the critical endogenous factor influencing the embryogenic response of microspores *in vitro*, it was to necessary to determine the late-uninucleate microspore stage related buds of Topas**.** We used Vernier caliper to measure the bud length and then put the bud on a glass slide with a drop of ddH_2O following gently squashing the bud by a glass rod. After that, the sample was observed under inverted microscopy (OLYMPUS IX71, Japan) in bright field. And the developmental stage of microspore was judged by its shape and the result of DAPI (4',6'-diamidino-2-phenylindole hydrochloride) staining. We found that the bud length region (3.5-4 mm) was highly correlated to the late-uninucleate microspores of Topas.

Microspore isolation and culture

The approximately 20 cm upper main stalks with inflorescences were nipped off the plants. They were covered by ice immediately to keep their viability. Then, the collected samples were quickly brought to the lab for buds selection. Only the 3.5-4 mm Topas buds were selected and kept. The selected buds were sterilized in Bus disinfectant for 15 min, rinsed 5 times in cooled sterile water for 1 min, and transferred to 50 ml disposable EP tubes that containing 5 ml filter sterilized 13% sucrose. Later, the buds were gently crushed by a sterilized glass rod. The slurry was passed through 30 μm mesh screens (Sefar, http://www.sefar.us/) to select the late-uninucleate stage microspores[1]. The filtrate was centrifuged at 1100 rpm for 4 min, and then the supernatant was discarded. After two-round washings by the fresh NLN-13 medium, the microspores were resuspended at a density of 40,000 per ml in NLN-13 medium. Aliquots of 5 ml suspension were plated in 9 cm Petri dishes for culture. The isolated microspores were divided into three parts: one was harvested immediately (0 h) and the other two were collected after 32℃ and 18℃ treatments for 6 h, respectively. Meanwhile, three Petri dishes of each sample were randomly chosen for photographing. Three or more micrographs from different microscopic view of each Petri dish were taken under inverted microscopy (OLYMPUS IX71, Japan). Then the diameters of microspores and the number of swollen microspores of each micrograph were measured and counted by using ImageJ software (http://rsb.info.nih.gov/ij/). These three collected samples were frozen in liquid nitrogen immediately and stored at -80℃ for subsequent extractions of genomic DNA and total RNA.

DAPI staining

The isolated microspores were fixed in Carnoy's Fluid (methanol: glacial acetic acid = 3: 1, volume ratio) at 4℃ in darkness overnight. Then the fixed samples were rinsed thrice by ddH_2O following DAPI (1μg/ml) (product no. 10236276001, Roche) staining at 4℃ in darkness for 3h.

Subsequently, the stained samples were placed on clean glass slides for observation with UV excitation under inverted microscopy (OLYMPUS IX71, Japan).

Genomic DNA and total RNA extractions

For genomic DNA extraction, one 5 mm stainless steel bead and 750 μl CTAB (hexadcetyltrimethylammouniumbromide) solution were added into each 2 ml safe-lock microtube that containing sample. Then the samples were homogenized on the high-throughput tissue lyser TL-2020 (Heros-Mole) for 38 seconds at 1800 rpm (Do not exceed this time as it may result in DNA shearing). The following procedure for extraction of genomic DNA was carried out according to the conventional CTAB protocol. After eliminating RNA contamination by RNase I digestion at 37℃ for 30 min, the quality and concentration of extracted DNA were determined by agarose gel electrophoresis and Qubit® 2.0 Fluorometer (Invitrogen, USA), respectively. The total RNA of each sample was extracted by using the EASYspin Plus Plant RNA Kit (Aidlab Biotech, Beijing, China). The yield and purity of RNA were detected by Qubit® 2.0 Fluorometer, and the intactness of RNA was verified by electrophoresis on a 0.8% agarose gel.

Genome-wide bisulphite sequencing

The qualified genomic DNA of each sample was sent to BGI (BGI Tech Solutions Co., Ltd, Shenzhen, China) for bisulfite sequencing with the following procedures: 3 μg genomic DNA were firstly fragmented by sonication instrument (Covaris). Then the 100-300 bp fragments were selected and purified with MiniElute PCR Purification Kit (QIAGEN). Subsequently, End Repair Mix was added to the purified fragments and

the mixture was incubated at 20℃ for 30 min. Then the reaction product was purified with MiniElute PCR Purification Kit (QIAGEN) and a single ‘A’ nucleotide was added to the 3’ ends of the blunt fragments. After repurification, the methylated adapters were added to both 5’ and 3’ ends of each strand of the genomic fragments. Later, the reaction product was separated by agarose gel electrophoresis and a narrow 300-400 bp size-range was selected and purified with QIAquick Gel Extraction Kit (QIAGEN) for subsequent bisulfite treatment by using the Methylation-Gold Kit (Zymo Research, Irvine, CA) following the manufacturer’s instructions. After conversion, the DNA was amplified by PCR for 10 cycles and the PCR product was separated by agarose gel electrophoresis. Then a narrow 350-400bp size-range was selected as libraries and purified with QIAquick Gel Extraction Kit (QIAGEN). A final control of quality and quantity of libraries was determined by an Agilent 2100 bioanalyzer instrument (Agilent DNA 1000 Reagents) and a real-time quantitative PCR (QPCR) (TaqMan Probe), respectively. The qualified libraries were amplified on cBot to generate the cluster on the flowcell (TruSeq PE Cluster Kit V3-cBot-HS, Illumina). Then the amplified flowcell will be sequenced pair end on the HiSeq 2000 System (TruSeq SBS KIT-HS V3, Illumina) and read length 90 bp was the sequencing strategy.

Genome-wide cytosine methylation analysis

The three data sets were analyzed based on the following steps: FastQC v0.11.2 (http://www.bioinformatics.bbsrc.ac.uk/projects/fastqc/) was executed on all .fq files in order to assess the basic quality control metrics (base quality distribution, GC content, relative abundance of each base at each read position). Each file containing the paired-end reads was filtered using an in-house Perl script with a minimum quality threshold of Q20 (98.5%). The filtered data was trimmed by Trimmomatic-0.33[2] with the following parameters: LEADING:3 TRAILING:3 SLIDINGWINDOW:4:15 MINLEN:80. Then the trimmed data was mapped to the released genome of *B. napus*

(Brassica_napus_v4.1.chromosomes.fa, deposited at http://www.genoscope.cns.fr/brassicanapus) by using Bismark v0.12.5[3] with the following parameters: --non_directional -n 1 -l 45. The generated SAM files were then fed into the deduplicate_bismark subprogram for de-duplication and the bismark_methylation_extractor subprogram to extract the methylations, which were stored in *.CX_report.txt files. The *.CX_report.txt file was subsequently separated according to CG, CHG, and CHH contexts and saved into three files in the format required by the methylKit v0.5.7[4]. After that, the split files were as input for methylKit to produce methylations. And bases with too low (< 10x) or too high coverage (bases that had more than the 99.9th percentile of coverage in each sample) were discarded. The DMRs were identified conservatively using q-value<0.01 and methylation difference>=25% (Supplementary Data S1). Then we employed the getAssociationWithTSS function of methylKit to get the distance information from DMRs to TSSs (Transcription Start Sites). And the TSSs with distance no more than 2kb upstream or downstream from the DMRs were selected. Thereafter, an in-house R script was used to retrieve the genes locus from Brassica_napus.annotation_v5.gff3 file (http://www.genoscope.cns.fr/brassicanapus/data) according to the selected TSSs position information, and these genes were considered as DRGs (Supplementary Data S3). Subsequent descriptive statistical results of the methylKit indicated that the CpG read coverage distribution of each sample exhibited only one good peak on the left side, which excluded the possibility of PCR duplication bias (Supplementary Fig. S1). Moreover, the % CpG methylation of each sample exhibited a classical bimodal distribution, indicating that the majority of bases had either high or low methylation levels (Supplementary Fig. S1). A scatter plots of the % methylation values for each pair of the three samples indicated that the CG methylation pattern exhibited the expected bimodal distribution, whereas CHH and CHG exhibited mostly unmethylated cytosines (Supplementary Fig. S2). The Pearson correlation coefficients between the three samples were high for the CG bases (0.98) (Supplementary Fig. S2). In addition, the identified mehtylated cytosines and DMRs were plotted by Circos (http://www.circos.ca/).

Summarizing the DMRs over pre-defined regions (i.e., promoters, exons, introns) was desirable and useful. Our results also demonstrated that the DMRs in all three pairwise samples were mainly distributed in intergenic and promoter regions; the fewest number of DMRs was observed in the introns (Supplementary Fig. S4).

Pyrosequencing validation

The primers for pyrosequencing validation were designed by PyroMark Assay Design 2.0 (Supplementary Table S11) and synthesized by BGI Company (BGI Tech Solutions Co., Ltd, Shenzhen, China). The following steps including bisulfite treatment on genomic DNA, purification of the treated DNA, PCR amplification, and pyrosequencing detection on PyroMark Q96 ID platform (QIAGEN) were performed by BGI Company according to their internal standard protocols.

Prediction of the CpG islands in the genome of *B. napus*

The CpG islands (CGIs) are thought to be sites with high incidences of DNA methylation/demethylation. To explore the relationships between DMRs and CGIs, we used CpG Island Searcher (cpgi130, http://cpgislands.usc.edu/download.htm) to predict the putative CGIs throughout the whole genome of *B. napus*. And a total of 68,090 (21,094 in the A genome and 46,996 in the C genome) putative CGIs were identified (Supplementary Table S3). The number of CGIs in the C genome was more than two-fold higher than that in the A genome, and the proportions of TL/TLC (total length of CGI/total length of chromosome) were 3.786% in the C genome and 2.957% in the A genome (Supplementary Table S3). Subsequently, we conducted CG DMR annotation in the CGIs and discovered that more DMRs were located in CGI shores compared with CGIs in all three pairwise samples (Supplementary Fig. S5).

Digital expression analysis

The total 502848 expressed sequence tags (ESTs) of *B. napus* were obtained from the NCBI website (ftp://ftp.ncbi.nih.gov/repository/UniGene/Brassica_napus/) as UniGene files (Bna.seq.all.gz). All the CDSs of the DMR-related genes were collected as a query file in fasta format. Then, local blastn program was executed based on the methods described preciously[5]. Eventually, the digital expression profile was suggested by analyzing the EST counts based on UniGene (http://www.ncbi.nlm.nih.gov/UniGene/UGOrg.cgi?TAXID=3708).

Supplementary References

1. Malik, M.R. *et al*. Transcript profiling and identification of molecular markers for early microspore embryogenesis in *Brassica napus*. *Plant Physiol.* **144,** 134–154 (2007).
2. Bolger, A.M., Lohse, M. & Usadel, B. Trimmomatic: a flexible trimmer for Illumina sequence data. *Bioinformatics* **30,** 2114–2120 (2014).
3. Krueger, F. & Andrews, S.R. Bismark: A flexible aligner and methylation caller for Bisulfite-Seq applications. *Bioinformatics* **27,** 1571–1572 (2011).
4. Akalin, A. *et al*. methylKit: A comprehensive R package for the analysis of genome-wide DNA methylation profiles. *Genome Biol.* **13,** R87 (2012).
5. Li, J. *et al*. Genome-wide survey and expression analysis of the putative non-specific lipid transfer proteins in *Brassica rapa* L. *PLoS ONE* **9,** e84556 (2014).

Supplementary Figures

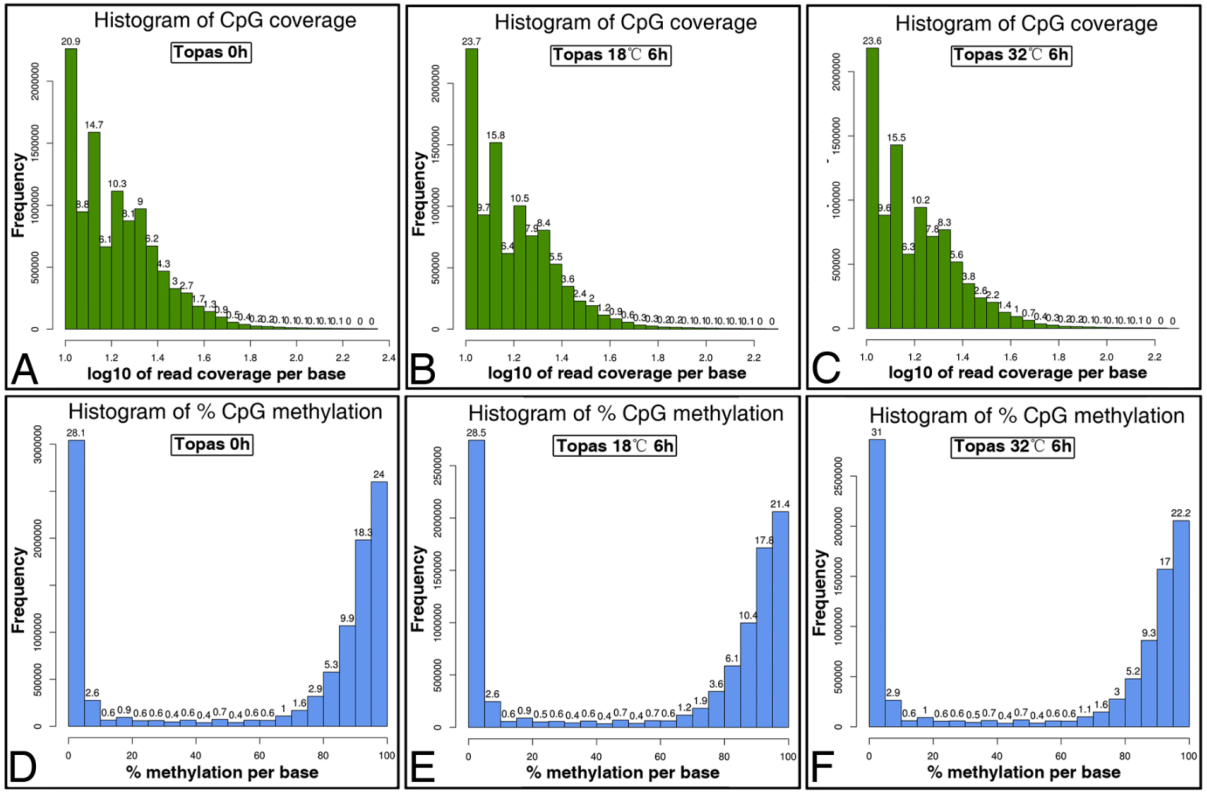

Supplementary Figure S1. The % methylation and read coverage per cytosine for each sample.

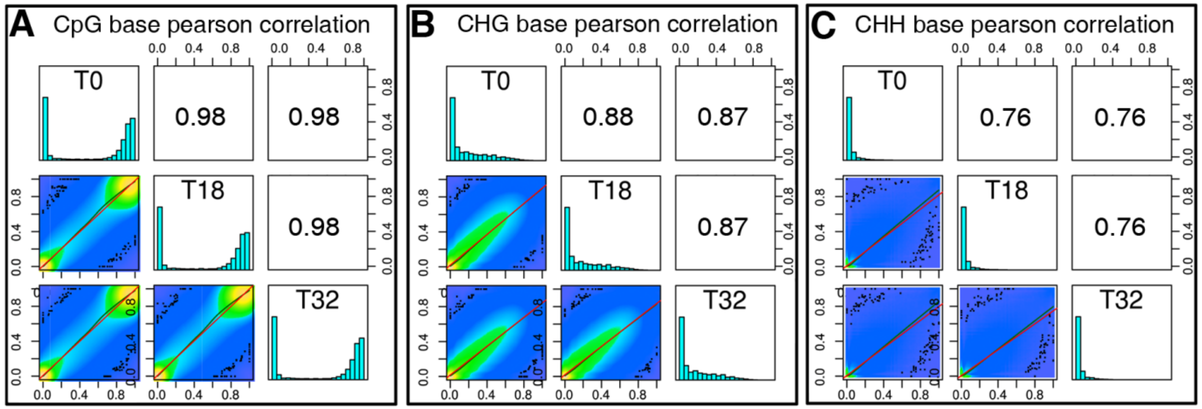

Supplementary Figure S2. Scatter plots of % methylation values for each pair in three samples. Numbers on upper right corner denote pairwise Pearson's correlation scores.

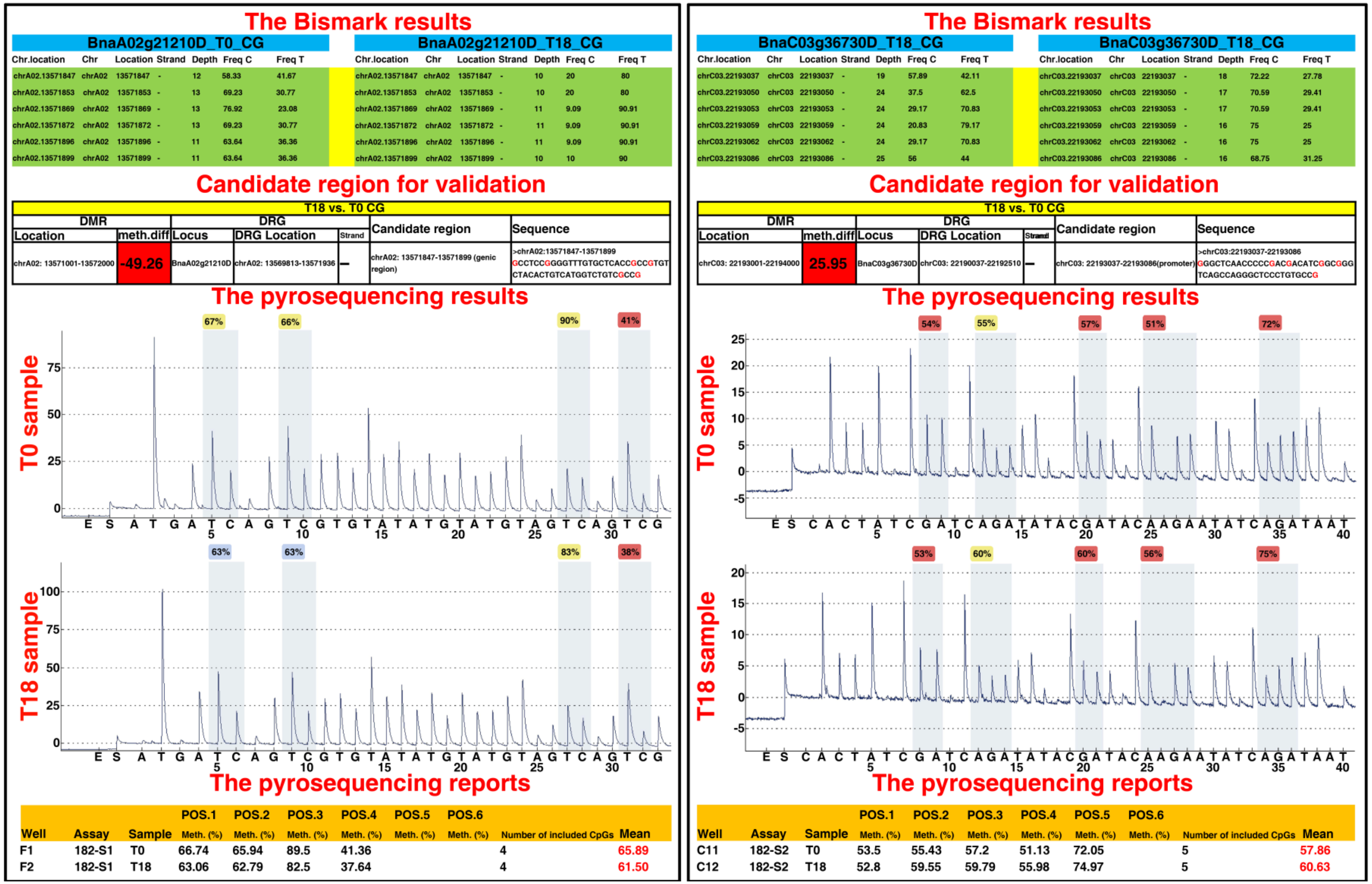

Supplementary Figure S3. Pyrosequencing validation of the two randomly selected DMRs.

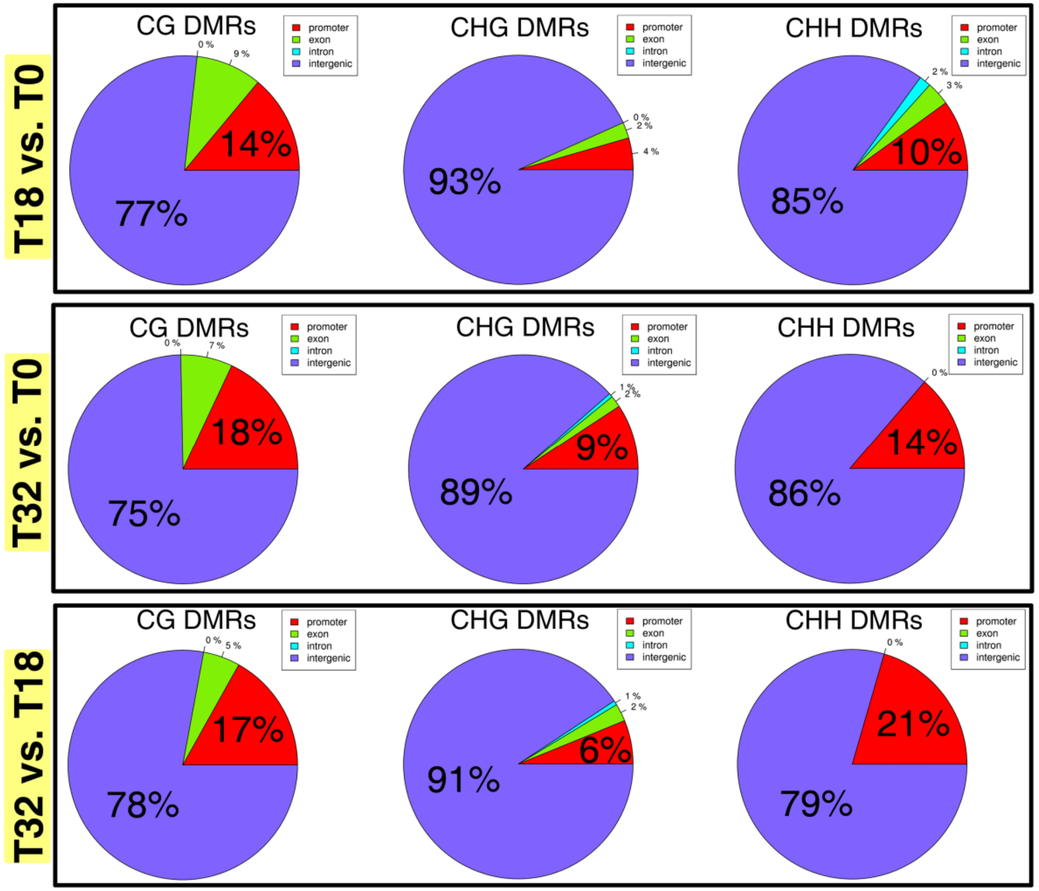

Supplementary Figure S4. The annotations of DMRs by promoters, introns, exons, and intergenic regions.

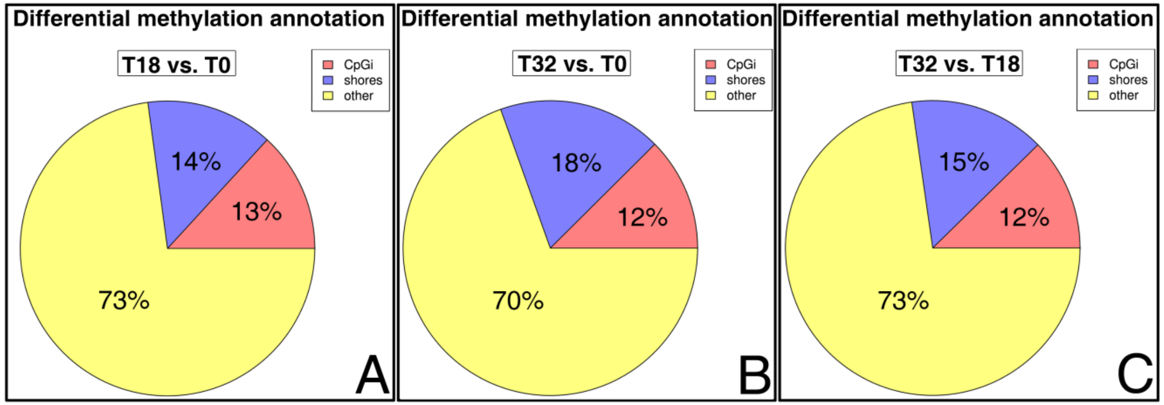

Supplementary Figure S5. Differential methylation annotations by CpG islands.

Supplementary Tables

Supplementary Table S1. The sequencing depth of each sample

Sample	Sequence pairs	Sequencing depth
T0	127433878	26.98
T18	129117125	27.33
T32	128794726	27.26

Supplementary Table S2. The Bismark reports for the sequencing data

Sample	UME[a] (%)	TNCA[b]	CpG			CHG			CHH		
			MC[c]	UC[d]	PMC[e] (%)	MC	UC	PMC (%)	MC	UC	PMC (%)
T0	49.4	2010617773	196236774	167839264	53.9	61756403	302788225	16.9	42592001	1239405106	3.3
T18	48.8	1925528501	182554979	159645903	53.3	58275451	288125115	16.8	44214612	1192712441	3.6
T32	47.8	1900255744	170962372	159684788	51.7	53164041	288595800	15.6	42888755	1184959988	3.5

[a]UME, Uniquely Mapping Efficiency; [b]TNCA, Total Number of Cytosines Analysed; [c]MC, Methylated Cytosines; [d]UC, Unmethylated Cytosines; [e]PMC, The Proportion of Methylated Cytosines.

Supplementary Table S3. The summarized information of the putative CpG islands in the genome of *B. napus*

Chromosome	NCpGi[a]	TL[b] (bp)	TLC[c]	TL/TLC (%)	Chromosome	NCpGi	TL (bp)	TLC	TL/TLC (%)
BnaA01	2064	696192	23267856	2.992	BnaC01	4800	1549082	38829317	3.989
BnaA02	2128	688961	24793737	2.779	BnaC02	5478	1734506	46221804	3.753
BnaA03	2718	922586	29767490	3.099	BnaC03	6454	2083225	60573394	3.439
BnaA04	1601	532683	19151660	2.781	BnaC04	5150	1704500	48930237	3.484
BnaA05	2010	687626	23067598	2.981	BnaC05	5534	1842609	43185227	4.267
BnaA06	2321	766034	24396386	3.140	BnaC06	4050	1423154	37225952	3.823
BnaA07	2014	680828	24006521	2.836	BnaC07	5105	1630025	44770477	3.641
BnaA08	1538	516746	18961941	2.725	BnaC08	4818	1581073	38477087	4.109
BnaA09	2723	915403	33865340	2.703	BnaC09	5607	1848634	48508220	3.811
BnaA10	1977	649714	17398227	3.734					
Total BnaA	21094	7056773	238676756	2.957	Total BnaC	46996	15396808	406721715	3.786

Selected lower limits: %GC=55, ObsCpG/ExpCpG=0.65, Length=200. [a]NCpGi, numbers of the CpG islands; [b]TL, total length of CpGi; [c]TLC, total length of chromosome.

Supplementary Table S4. The number of hypermethylated and hypomethylated transposons in DMRs.									
**Type**	**CG DMR**			**CHG DMR**			**CHH DMR**		
	**T18 vs. T0**	**T32 vs. T0**	**T32 vs. T18**	**T18 vs. T0**	**T32 vs. T0**	**T32 vs. T18**	**T18 vs. T0**	**T32 vs. T0**	**T32 vs. T18**
hypermethylated transposon	11	22	19	28	9	14	3	8	2
hypomethylated transposon	18	50	32	24	85	66	1	0	4
Total differential methylation of transposons	29	72	51	52	94	80	4	8	6

Supplementary Table S5. The number of each type of TE in DMRs

Transposon categories	CG DMR			CHG DMR			CHH DMR		
**Retrotransposons**	**T18 vs. T0**	**T32 vs. T0**	**T32 vs. T18**	**T18 vs. T0**	**T32 vs. T0**	**T32 vs. T18**	**T18 vs. T0**	**T32 vs. T0**	**T32 vs. T18**
LINE	12	37	28	18	23	25	1	0	1
LTR	0	0	0	0	0	0	0	0	0
LTR/Caulimovirus	0	1	0	0	0	0	0	0	0
LTR/Copia	4	12	6	6	21	14	3	4	0
LTR/ERV	0	0	0	0	0	0	0	0	0
LTR/Foamy	0	0	0	0	0	0	0	0	0
LTR/Gypsy	3	7	10	12	19	18	0	2	0
LTR/Pao	0	1	0	0	0	0	0	0	0
Total Retrotransposons	19	58	44	36	63	57	4	6	1
**DNA-transposons**									
DNA/Academ	0	0	0	0	0	0	0	0	0
DNA/CMC-EnSpm	1	4	1	5	11	7	0	0	1
DNA/Ginger	0	0	0	0	0	0	0	0	0
DNA/hAT-Ac	0	0	0	3	1	0	0	0	0
DNA/hAT-Tag1	0	2	0	1	0	0	0	0	0
DNA/hAT-hATw	0	0	0	0	0	0	0	0	0
DNA/hAT-Tip100	1	0	0	0	0	2	0	0	0
DNA/MuLE-MuDR	3	2	0	6	11	8	0	1	0
DNA/P	0	0	0	0	1	0	0	0	0
DNA/PIF-Harbinger	2	3	3	1	5	4	0	1	4
DNA/TcMar-Stowaway	0	0	0	0	0	0	0	0	0
DNA/TcMar-Tc1	0	0	0	0	0	0	0	0	0
RC/Helitron	3	3	3	0	2	2	0	0	0
DNA/unknown	0	0	0	0	0	0	0	0	0
Total DNA-transposons	10	14	7	16	31	23	0	2	5

Supplementary Table S6. The distribution of DMTs on each chromosome

Chromosome	CG DMT			CHG DMT			CHH DMT		
	T18 vs. T0	T32 vs. T0	T32 vs. T18	T18 vs. T0	T32 vs. T0	T32 vs. T18	T18 vs. T0	T32 vs. T0	T32 vs. T18
chrA01	0	0	0	2	4	2	0	0	0
chrA02	1	3	0	0	1	2	0	0	0
chrA03	2	3	1	2	1	3	0	0	0
chrA04	0	3	1	1	2	2	0	0	0
chrA05	2	4	0	1	5	4	0	1	0
chrA06	3	5	0	2	4	7	0	1	0
chrA07	0	3	2	1	3	3	0	0	0
chrA08	0	1	2	4	1	0	0	0	0
chrA09	1	4	2	1	3	3	0	0	0
chrA10	0	0	1	2	1	1	0	0	0
Total in A genome	9	26	9	16	25	27	0	2	0
chrC01	6	5	5	8	6	11	0	0	0
chrC02	2	4	0	6	11	8	0	1	0
chrC03	2	2	4	4	5	7	0	0	1
chrC04	1	10	6	2	3	3	1	0	0
chrC05	2	8	7	3	10	2	0	1	0
chrC06	3	6	4	3	6	6	1	1	3
chrC07	0	6	4	3	7	11	2	2	2
chrC08	1	2	2	1	7	2	0	0	0
chrC09	3	3	10	6	14	3	0	1	0
Total in C genome	20	46	42	36	69	53	4	6	6

Supplementary Table S7. The distribution of DRGs on each chromosome of *B. napus*

Chromosome	CG DRG			CHG DRG			CHH DRG		
	T18 vs. T0	T32 vs. T0	T32 vs. T18	T18 vs. T0	T32 vs. T0	T32 vs. T18	T18 vs. T0	T32 vs. T0	T32 vs. T18
chrA01	1	1	1	0	1	1	0	0	0
chrA02	2	5	1	0	3	1	0	0	0
chrA03	6	6	8	2	0	0	1	2	0
chrA04	2	1	2	1	0	1	1	0	0
chrA05	0	2	2	0	2	0	0	0	0
chrA06	1	2	0	0	2	2	0	0	0
chrA07	0	0	2	0	1	0	1	0	0
chrA08	0	1	3	0	1	1	0	0	0
chrA09	1	5	2	0	1	1	0	0	1
chrA10	1	1	0	0	2	0	1	0	0
Total in A genome	14	24	21	3	13	7	4	2	1
chrC01	3	4	4	0	3	4	1	1	2
chrC02	0	0	0	1	0	2	1	0	1
chrC03	4	7	3	0	3	7	0	2	0
chrC04	2	7	3	0	0	0	0	0	0
chrC05	2	6	2	1	2	1	1	0	3
chrC06	1	1	2	0	1	2	1	1	1
chrC07	2	1	3	0	1	1	1	2	1
chrC08	1	4	2	0	3	1	0	1	0
chrC09	4	2	1	3	5	1	2	0	1
Total in C genome	19	32	20	5	18	19	7	7	9

Supplementary Table S8. The total number of each kind of DRG in three pairwise comparisons			
**Type**	**T18 vs. T0**	**T32 vs. T0**	**T32 vs. T18**
CG DRG	33	56	41
CHG DRG	8	31	26
CHH DRG	11	9	10
Total	52	96	77

Supplementary Table S9. The number of DRGs distributed on each CCB of *B. napus*										
Block	CG DRG			CHG DRG			CHH DRG			total DRGs in each block
	T18 vs. T0	T32 vs. T0	T32 vs. T18	T18 vs. T0	T32 vs. T0	T32 vs. T18	T18 vs. T0	T32 vs. T0	T32 vs. T18	
A	1	6	2	0	1	1	0	0	0	11
B	0	2	3	0	0	0	0	0	0	5
C	0	0	1	0	2	1	1	0	1	6
D	1	2	1	0	1	1	1	0	0	7
E	0	3	1	0	2	2	0	0	0	8
F	3	4	4	1	3	2	1	1	1	20
G	0	0	0	0	0	0	0	0	0	0
H	0	0	0	0	0	0	0	0	0	0
I	1	1	3	0	0	0	0	0	0	5
J	2	3	4	0	0	2	1	0	0	12
K	0	0	0	0	0	0	0	0	0	0
L	0	1	0	0	0	0	0	0	0	1
M	2	1	1	0	3	2	0	0	0	9
N	0	3	1	0	2	1	0	1	1	9
O	1	0	0	0	1	1	0	0	1	4
P	0	0	0	0	2	0	0	0	0	2
Q	1	1	0	1	0	0	0	0	0	3
R	1	3	0	2	2	4	2	0	0	14
S	0	0	0	0	0	0	0	0	0	0
T	1	1	1	0	1	0	0	0	0	4
U	4	3	4	0	2	6	2	2	3	26
V	0	1	0	0	1	0	0	0	0	2
W	0	0	0	0	2	1	1	0	1	5
X	1	2	1	0	1	0	1	1	0	7

Supplementary Table S10. Primers used in quantitative RT-PCR

Gene	Forward Primer	Reverse Primer
BnaA09g40710D	5' GGTTGATGATGATGATGATGATGATAAT 3'	5' GCGGAACCACCTTGATCACTCT 3'
BnaC05g11580D	5' GGTTGACGACTTTCTTCTGGTTTGGTGA 3'	5' GTAGCTGGCATTCATTCACACACGTCTC 3'
BnaC05g29060D	5' CAGGACAGGGTGGTGGTAATTTT 3'	5' TTCAACTCACAGAAAAAATAATCCAAATA 3'
BnaA05g23830D	5' CACGTCCGTCACGGGTTCACACCT 3'	5' CCCATTGTCAACATCGCGAAGCTCTAC 3'
BnaC09g48500D	5' CTTGCTGGCTTTTTTTTTGTGGAACTTT 3'	5' AAACGCTGGCAAAGAAACTGAACACAT 3'
BnaA04g18120D	5' AGGATGACAGCGAAGAAGACGAAGATG 3'	5' CCAAAAACCTCAGTGTTCTTCCATGATG 3'
BnaA03g02950D	5' CTTTAACTCTTTTGCTCTCTTTGGTGAT 3'	5' TCCCCTTTCCTTCAACGTCTTTAC 3'
BnaC03g58470D	5' GTCAGGGGACAAACAACTTCTTGGCTCA 3'	5' AAAATGAGCAGCACCACACAAGGAGACA 3'
BnaA05g05760D	5' GGACTTGGTGCTGAGGTGGGGATAA 3'	5' AAAGCCCATCCTCTCCTAGTCTCCACAG 3'
BnaA02g14590D	5' AGCAAGTAGCAAGTCATGACTTCTTCCTC 3'	5' GCAGAGACAGCGGGAGGATACG 3'
BnaC05g43880D	5' GCCCGGTTTAGATTGGTTATTATTTT 3'	5' AAAAAAAAAGAACAATCTGAGGACTAAAGAT 3'
BnaA09g41410D	5' CGATGAAGAACCGACGACAACC 3'	5' TCTGCTTCATTACTCCGGGCTTC 3'
BnaC02g00380D	5' GCGCGATTCATGCTAAGAGGG 3'	5' TAAACAGATCAAGAACTAAACACGAGCC 3'
BnaA08g15210D	5' AACAAGACACACGCACAATACACATG 3'	5' GCAAACCAAACAGTCCAAAGCTATCT 3'
BnaC06g24840D	5' AGAGGAAGTGGACATGACGACGATAAAG 3'	5' CGACTGTACACTGGTTCGGTTGACAA 3'
BnaA09g32770D	5' CAGGGTGAACAAAAAAATGAGCG 3'	5' CAGGCCGAAGATACAAGAAGCAAA 3'
β-actin	5' GAGCTCCGTGTTGCCCCTGAAGA 3'	5' TGGATAGCAACATACATGGCAGGGACA 3'

Supplementary Table S11. The primers for pyrosequencing validation

Primer name	Primer sequence	5' modification
chrA02:13571847-13571899-Forward Primer	5' ATTGAGGTAGTTTTTAGTAATTATAATGAT 3'	
chrA02:13571847-13571899-Reverse Primer	5' AACTCCAACCACCACTAATTCACTTT 3'	5' Biotin
chrA02:13571847-13571899-Sequencing Primer	5' GTTTTAGGGGTTTGTG 3'	
chrC03:22193037-22193086-Forward Primer	5' AGAGATTTTTGGTAGGTTTTATTTAAAG 3'	5' Biotin
chrC03:22193037-22193086-Reverse Primer	5' CCCATACATAAAATTCCACTCTCA 3'	
chrC03:22193037-22193086-Sequencing Primer	5' TTAAAATATCCACACAAAAAACCCT 3'	
